# Katalyst: knowledge-guided semantic alignment via contrastive learning for enzyme turnover prediction

**DOI:** 10.1093/bioinformatics/btag709

**Published:** 2026-10-03

**Authors:** Ruilin Li, Senyu Tang, Jiaqi Deng, Taixing Qiu, Fei Guo, Jijun Tang, Xiaoyi Liu

**Affiliations:** College of Computer Science and Software Engineering, Shenzhen University, Shenzhen, 518060, China; School of Computer Science and Control Engineering, Shenzhen University of Advanced Technology, Shenzhen, 518107, China; College of Computer Science and Software Engineering, Shenzhen University, Shenzhen, 518060, China; Department of Computer Science and Software Engineering, Southern University of Science and Technology, Shenzhen, 518055, China; School of Computer Science, Nanjing University, Nanjing, 210023, China; School of Computer Science and Engineering, Central South University, Changsha, 410083, China; School of Computer Science and Control Engineering, Shenzhen University of Advanced Technology, Shenzhen, 518107, China; School of Chinese Materia Medica, Beijing University of Chinese Medicine, Beijing, 102488, China

## Abstract

**Motivation:**

The turnover number ($k_{\text{cat}}$) is crucial for understanding enzyme kinetics, yet its experimental determination remains costly and low-throughput. Existing deep learning approaches often rely on single-substrate assumptions and overlook full reaction contexts or language-informed semantic representations, limiting their accuracy and generalization across diverse reactions.

**Results:**

To address these challenges, we propose Katalyst, a knowledge-guided semantic alignment framework for $k_{\text{cat}}\;\text{prediction}$ based on contrastive learning. Katalyst integrates enzyme sequences, protein textual descriptions, substrates and products into a shared latent space using multi-channel encoders and contrastive alignment. An interaction-aware fusion module is designed to capture complementary cross-modal information. Experimental results demonstrate that Katalyst achieved the best overall performance, with the RMSE of 0.9880, $R^{2}$ of 0.5829, and SRCC of 0.7603. Compared with the State-Of-The-Art (SOTA) baselines, Katalyst reduced RMSE by 3.01% and improved $R^{2}$ and SRCC by 4.72% and 2.31%, respectively. Moreover, ablation studies and evaluations across sequence scales further confirmed the effectiveness of semantic alignment and multimodal feature fusion. Overall, Katalyst provides a robust and accurate framework for $k_{\text{cat}}\;\text{prediction}$.

**Availability and implementation:**

The code and data are available at https://github.com/liruilin-SUAT/Katalyst.

## 1 Introduction

Enzymes are the fundamental catalysts of life, orchestrating diverse biochemical transformations by lowering activation energy barriers and enabling physiological reactions (Wolfenden and Snider 2001, Zhou *et al.* 2026), a capability that exerts a profound impact on enzyme evolution, metabolic engineering, and synthetic biology (Kuchner and Arnold 1997, Currin *et al.* 2015, Sánchez *et al.* 2017, Kang *et al.* 2025, Liu *et al.* 2025, 2026, Wang and Zheng 2026). The key element of enzyme kinetics is the number of turnover ($k_{\text{cat}}$), which defines the maximum number of substrate molecules converted into product by a single active enzyme site per unit of time (Briggs and Haldane 1925). Accurate determination of $k_{\text{cat}}$ is essential for understanding metabolic flux (Davidi *et al.* 2016), proteome allocation (Sánchez *et al.* 2017), and cellular physiology, as well as for industrial applications such as metabolic engineering (Li *et al.* 2022a; Xu *et al.* 2026) and drug discovery (Liu *et al.* 2023, Srinivasan 2023, Ai *et al.* 2024).

Despite its importance, experimental $k_{\text{cat}}$ determination remains a major bottleneck, severely constrained by high costs, intensive labor, and low throughput (Bar-Even *et al.* 2011). This has created a stark data disparity. While the UniProt Knowledgebase (UniProtKB) (Magrane and UniProt Consortium 2011) database now hosts over 248 million protein sequences (Hu *et al.* 2025), repositories of experimentally measured kinetic parameters, such as BRENDA (Schomburg *et al.* 2017) and SABIO-RK (Wittig *et al.* 2018), contain tens of thousands of experimentally measured $k_{\text{cat}}$ values (Yu *et al.* 2023). Furthermore, non-standardized assay conditions across laboratories introduce substantial noise into datasets, complicating cross-organism kinetic comparisons (Wittig *et al.* 2018).

To address this kinetic gap, deep learning has emerged as a powerful tool, redefining $k_{\text{cat}}$ prediction as a compound-protein interaction problem. For example, DLKcat (Li *et al.* 2022a) integrated Graph Neural Network (GNN) (Scarselli *et al.* 2009) and Convolutional Neural Networks (CNNs) (Krizhevsky *et al.* 2012) to predict $k_{\text{cat}}$ across diverse organisms based on protein sequences and substrate structures. TurNup (Kroll *et al.* 2023) introduced a species-independent approach using differential reaction fingerprints and retrained ESM-1b (Rives *et al.* 2021) to represent complete chemical transformations. UniKP (Yu *et al.* 2023) further advanced the field by leveraging pre-trained ProtT5 language models (Elnaggar *et al.* 2022) and ensemble learning to systematically incorporate environmental factors like pH and temperature. For the specific task of mutant enzyme prediction, EITLEM-Kinetics (Shen *et al.* 2024) employed an ensemble iterative transfer learning strategy combined with the ProMolAtt and AttentionAgg module to capture adaptive interactions across multiple modalities. To quantify thermal dependencies, DLTKcat (Qiu *et al.* 2023) utilized Graph Attention Networks (Veličković *et al.* 2017) and a bidirectional attention mechanism (Li *et al.* 2022b) to refine interaction representations between compounds and proteins that are sensitive to temperature. MMKcat (Sun *et al.* 2025) introduced a robust multimodal architecture encoding enzyme sequences, substrates, products, and protein 3D structures within a unified framework, while further employing a prior-guided non-uniform mask training mechanism to ensure accurate $k_{\text{cat}}$ prediction even when 3D structural or product information is unavailable during inference. However, most existing methods primarily model enzyme and single substrate relationships based on sequence features, while overlooking the reaction level context, resulting in suboptimal performance (Sun *et al.* 2025).

To address these challenges, we propose a knowledge-guided semantic alignment framework for $k_{\text{cat}}$ prediction based on contrastive learning, named Katalyst. Katalyst learns a unified latent representation by aligning heterogeneous modalities, including enzyme sequences, protein text descriptions, as well as substrates and products represented by the Simplified Molecular Input Line Entry System (SMILES) (Weininger 1988). To integrate heterogeneous modalities, we used multi-stage contrastive alignment followed by interaction-based fusion. Under a late fusion scheme, the fusion module combines Multi-Head Attention (MHA) (Vaswani *et al.* 2017) and CNN architectures to capture complex cross-modal relationships. Finally, the fused representations are utilized for accurate $k_{\text{cat}}$ prediction through a regression head. Overall, the main contributions can be summarized as follows:

1. We propose a contrastive alignment strategy that projects heterogeneous modalities, including enzyme sequences, protein text descriptions, and SMILES representations of substrates and products, into a unified latent space, effectively bridging modality gaps and enhancing representation consistency.
2. We develop an interactive fusion framework that leverages attention mechanisms and CNNs to integrate cross-modal features, capturing complex cross-modal interactions and enhancing model robustness.
3. Katalyst outperforms the State-Of-The-Art (SOTA) baselines on benchmark datasets, and evaluations across sequence scales further demonstrate its effectiveness and robustness in $k_{\text{cat}}$ prediction.

## 2 Methods

### 2.1 Overall framework

In this study, we introduce Katalyst (as shown in Fig. 1), a novel knowledge-guided semantic alignment framework based on contrastive learning for $k_{\text{cat}}$ prediction. Katalyst exploits complementary biological knowledge from protein functional descriptions, enzyme sequences, and molecular structures to learn informative multimodal representations. Specifically, the framework consists of four main components, comprising a multichannel feature encoder, a contrastive representation alignment module, an interaction-driven fusion module, and a dual-branch predictor. Instead of relying solely on protein sequences, Katalyst incorporates functional descriptions as complementary biological knowledge. Frozen pre-trained encoders equipped with trainable adapters project heterogeneous modalities into a unified latent space while preserving their domain-specific knowledge. To bridge the semantic gap across modalities, a multi-stage contrastive learning strategy progressively aligns protein sequence representations with functional descriptions and subsequently with substrate and product representations. The aligned representations are then integrated through an interaction-driven fusion module that captures complementary cross-modal information via attention-based feature interaction. To improve robustness under incomplete molecular information, stochastic modality masking is employed during fusion and complemented by an auxiliary branch with deterministic masking for additional supervision. Finally, the fused representation is fed into a regression head to predict $k_{\text{cat}}$.

**Figure 1:**
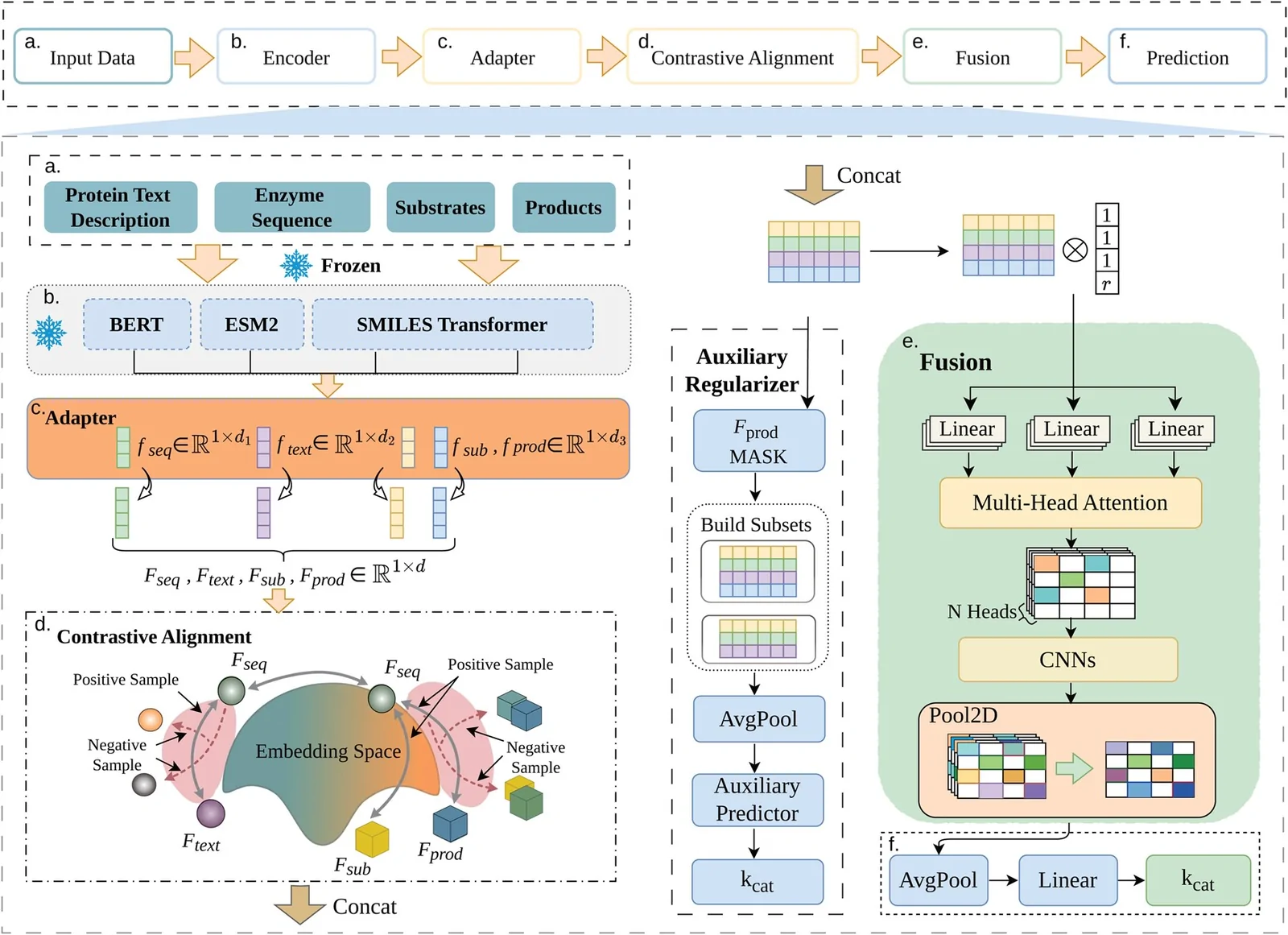
The overall framework of Katalyst for $k_{\text{cat}}$ prediction. The model consists of four primary stages: (b) Feature Encoder: Multimodal inputs, including protein descriptions, enzyme sequences, as well as combined substrates and products, are encoded by PubMedBERT, ESM2, and specialized Transformers, respectively. (c) Adapter and (d) Contrastive Alignment: Features are projected into a joint latent space via adapters, where contrastive alignment is applied to bridge the semantic gap using InfoNCE loss. (e) Feature Fusion: A fusion module incorporating MHA, CNNs, and pooling layers integrates the aligned features. (f) $k_{\text{cat}}$ Prediction: A final linear predictor are employed to estimate the $k_{\text{cat}}$ value.

### 2.2 Problem formulation

The reaction space catalyzed by enzymes is conceptualized as a network of complex interactions among enzymes, substrates, and products. The primary objective is to learn a mapping function f:X→y, where $y=log_{10}(k_{\text{cat}})$ value. The input space X is defined as a multimodal tuple (Q,T,S,P), comprising the enzyme sequence *Q*, functional text *T*, substrate structure *S*, and product structure *P*, respectively.

Specifically, the enzyme protein sequence of length $L_{e}$ is denoted as $Q=\{q_{1},q_{2},\ldots ,q_{L_{e}}\}$, where each $q_{i}$ denotes an individual amino acid residue. The textual modality is represented as $T=\{t_{1},t_{2},\ldots ,t_{L_{t}}\}$, where each $t_{i}$ denotes an individual text token and $L_{t}$ represents the total sequence length. To precisely accommodate reactions with multiple participants, the chemical entities are represented as collections of SMILES notations. The substrate and product sets are formalized as $S=\{S^{\left(1\right)},S^{\left(2\right)},\ldots ,S^{\left(n\right)}\}$ and $P=\{P^{\left(1\right)},P^{\left(2\right)},\ldots ,P^{\left(m\right)}\}$, where *n* and *m* are the number of substrates and products, respectively. Each individual substrate $S^{\left(i\right)}$ and product $P^{\left(j\right)}$ is formalized as a sequence of chemical characters with length $L_{s,i}$ and $L_{p,j}$, respectively. Here, the first subscript indicates the molecule modality (*s* for substrate, *p* for product) and the second signifies the index of the substrate or product. Based on these definitions, the task is framed as predicting the numerical $k_{\text{cat}}$ from the multimodal tuple (Q,T,S,P).

### 2.3 Feature encoder

#### 2.3.1 Protein sequence encoder

For enzyme protein sequences, we employed the pre-trained ESM2 model (specifically esm2_t33_650M_UR50D) (Lin *et al.* 2023) to extract high-dimensional biological embeddings. Given a sequence *Q* of length $L_{e}$, the model generates per-residue representations $f_{\text{seq}}^{\text{pos}}\in R^{L_{e}\times d_{1}}$ from its 33rd transformer layer, where $d_{1}$ denotes the latent dimension of ESM2. To obtain a global enzyme feature $f_{\text{seq}}\in R^{1\times d_{1}}$, mean pooling is performed across the residue tokens, excluding non-biological special tokens, defined as:


(1) $$f_{\text{seq}}=\frac{1}{L_{e}}\sum_{i=1}^{L_{e}}f_{\text{seq}}^{\text{pos}\;}[i].$$

#### 2.3.2 Textual description encoder

To incorporate domain-specific functional semantics, the pre-trained PubMedBERT (Gu *et al.* 2022) was leveraged to encode the textual description *T*. The model transforms the sequence into a hidden state matrix $H_{T}={\left[h_{1},h_{2},\ldots ,h_{L_{t}}\right]}^{⊤}\in R^{L_{t}\times d_{t}}$, where $d_{t}$ is the hidden dimension of PubMedBERT. To derive the final global feature $f_{\text{text}}\in R^{1\times d_{2}}$, where $d_{2}=2d_{t}$, we implemented a mixed readout strategy that concatenates the mean and standard deviation of these hidden states across all valid tokens:


(2) $$f_{\text{text}}=\mathrm{Concat}(\text{mean}(H_{T}),\text{std}(H_{T})).$$

#### 2.3.3 SMILES encoder

To capture the latent features of chemical participants, we employed a pre-trained SMILES Transformer (Honda *et al.* 2019) as the molecular encoder. Aiming to preserve comprehensive information for complex reactions, we retained all substrates and products without simplification. Formally, the aggregate substrate and product representations ($f_{\text{sub}},f_{\text{prod}}\in R^{1\times d_{3}}$, where $d_{3}$ denotes the latent dimensionality of molecular encoder) are derived by averaging the constituent participant embeddings within their respective sets:


(3) $$\begin{array}{cc} & f_{\text{sub}}=\frac{1}{n}\sum_{i=1}^{n}\;\text{SMILES}\_\text{Transformer}(S^{\left(i\right)}),\\ & f_{\text{prod}}=\frac{1}{m}\sum_{j=1}^{m}\;\text{SMILES}\_\text{Transformer}(P^{\left(j\right)}), \end{array}$$

where *n* and *m* denote the number of molecules in S and P. In cases where products are absent (m=0), $f_{\text{prod}}$ is initialized as a zero vector 0.

### 2.4 Feature adaptor

To bridge the dimensional discrepancy between the heterogeneous encoders and facilitate multi-modal fusion, we employed a set of task-specific adapter layers. These layers project the initial features $f_{\text{seq}}$, $f_{\text{text}}$, $f_{\text{sub}}$, and $f_{\text{prod}}$ into a unified latent space $R^{1\times d}$, where *d* denotes the target dimensionality of the unified latent space. Formally, the aligned representations are derived as follows:


(4) $$\begin{array}{cc} & F_{k}=\text{Linear}(\text{Dropout}(\text{ReLU}({\;\text{Linear}}_{k}(f_{k})))),\\ & k\in \{\text{seq},\;\text{text},\;\text{sub},\;\text{prod}\}. \end{array}$$

### 2.5 Feature alignment

To refine cross-modal semantic consistency, we introduced a contrastive alignment module leveraging the InfoNCE loss (Oord *et al.* 2018) to pull corresponding modal pairs closer in the latent space while pushing mismatched pairs apart. For a given batch of size *B*, let $\left(u_{i},v_{i}\right)$ denote the aligned feature pair of the *i*-th sample, where i∈{1,2,…,B}. The contrastive objective for a specific positive pair $\left(u_{i},v_{i}\right)$ relative to the remaining batch components is formulated as:


(5) $$\text{InfoNCE}(u_{i},v_{i})=-\text{log}\;\frac{\;\text{exp}(u_{i}{\;}^{⊤}v_{i}/\tau )}{\sum_{k=1}^{B}\;\text{exp}\;(u_{i}{\;}^{⊤}v_{k}/\tau )},$$

where τ is a temperature hyperparameter, and *B* is the batch size. As illustrated in the architecture, the alignment is divided into two primary objectives, including sequence-text alignment and sequence-chemical alignment.

Firstly, the sequence-text alignment ($L_{1}$) aligns functional natural language descriptions with their corresponding biological sequences. Within each mini-batch, features derived from the same enzyme record $\left(F_{\text{text},i},F_{\text{seq},i}\right)$ form a positive pair, while all mismatched combinations $\left(F_{\text{text},i},F_{\text{seq},k}\right)$, where k≠i, serve as negative instances. The resulting contrastive loss is formulated as:


(6) $$L_{1}=\frac{1}{B}\sum_{i=1}^{B}\text{InfoNCE}(F_{\text{text},i},F_{\text{seq},i}).$$

Furthermore, to bridge the modality gap, we aligned enzyme sequence representations with their corresponding substrate and product embeddings as sequence-chemical alignment ($L_{2}$). Within each mini-batch, a positive triplet is formed by the co-occurring sequence, substrate set, and product set from the same valid reaction entry, while all mismatched combinations serve as negative alternatives. The alignment loss is formulated by averaging these cross-modal contrastive components:


(7) $$L_{2}=\frac{1}{2B}\sum_{i=1}^{B}[\text{InfoNCE}(F_{\text{seq},i},F_{\text{sub},i})+\text{InfoNCE}(F_{\text{seq},i},F_{\text{prod},i})].$$

Therefore, the total alignment loss $L_{\mathrm{align}}$ is defined as the weighted sum of these components:


(8) $$L_{\mathrm{align}}=L_{1}+L_{2}.$$

### 2.6 Multi-modal fusion and prediction

Following the dimensional alignment via adapters and semantic refinement through contrastive alignment, the features of individual modalities $\{F_{\text{sub}},F_{\text{seq}},F_{\text{text}},F_{\text{prod}}\}\subset R^{1\times d}$ are integrated to capture complex cross-modal interactions.

For feature aggregation, the aligned features are concatenated to construct a multimodal context tensor. To enhance model robustness against missing product information, product features are subjected to a Bernoulli masking operation: $F_{\text{prod}}^{\prime }=F_{\text{prod}}⊙r$, where r∼Bernoulli(0.5). The aggregated input $X_{\text{fusion}}\in R^{4\times d}$ is formed as:


(9) $$X_{\text{fusion}}=\text{Stack}(F_{\text{sub}},F_{\text{seq}},F_{\text{text}},F_{\text{prod}}^{\prime }).$$

For attentive interaction, the aggregated tensor $X_{\text{fusion}}$ is fed into a MHA module to compute intermodal dependencies. The MHA mechanism dynamically weighs the mutual contributions of biological, chemical, and textual tokens across different representation subspaces:


(10) $$H_{\text{attn}}=\text{MultiHead}(X_{\text{fusion}}),$$

where $H_{\text{attn}}\in R^{n_{\text{head}}\times 4\times d}$ represents the enriched multi-head attention maps, with $n_{\mathrm{head}}$ denoting the number of attention heads.

Moreover, for hierarchical readout, to extract local patterns and reduce dimensionality, $H_{\text{attn}}$ is processed through CNNs followed by a 2D pooling layer (Pool2D):


(11) $$Z=\text{Pool}2D\left(\text{CNNs}(H_{\text{attn}})\right),$$

where $Z\in R^{4\times d}$ represents the refined cross-modal feature matrix. Finally, the comprehensive representation is passed through a 1D pooling layer (AvgPool) and a linear output head to predict the $k_{\text{cat}}$ value:


(12) y=Linear(AvgPool(Z)),

where *y* represents the predicted $k_{\text{cat}}$ value.

### 2.7 Training objectives and regularization

To improve robustness and cross-modal synergy, Katalyst is optimized through a multitask learning framework. The contrastive alignment module (Section 2.5) serves as a semantic constraint to align biological and chemical representations, providing more consistent features for multimodal fusion. However, missing modalities can substantially degrade multimodal learning performance (Ma *et al.* 2022). To address incomplete reaction information, we introduced an auxiliary regularization module (Sun *et al.* 2025). Specifically, Bernoulli masking (Section 2.6) was employed to simulate missing product scenarios, generating multimodal feature subsets as:


(13) $$M\in \{{\left(F_{\text{sub}},F_{\text{seq}},F_{\text{text}},F_{\text{prod}}\right)}^{⊤},{\left(F_{\text{sub}},F_{\text{seq}},F_{\text{text}},0\right)}^{⊤}\},$$

where 0 represents the zero vector with the same dimension as $F_{\text{prod}}$. The regularization loss $L_{\text{aux}}$ minimizes prediction discrepancies between masked subsets and the ground-truth kcat values:


(14) $$L_{\text{aux}}=\frac{1}{B}\sum_{j=1}^{B}\sum_{i=1}^{N}{\left(\hat{y_{j}}-\Phi_{\text{aux}}(\mathrm{AvgPool}(M_{j,i}))\right)}^{2},$$

where *N* denotes the number of generated feature subsets (N=2), $\Phi_{\text{aux}}$ represents the auxiliary predictor, and $\hat{y_{j}}$ represents the ground-truth $k_{\text{cat}}$ value of the *j*-th sample. The overall objective is defined as:


(15) $$\begin{array}{cc} & L_{\text{pred}}=\frac{1}{B}\sum_{j=1}^{B}{\left(\hat{y_{j}}-y_{j}\right)}^{2},\\ & L_{\text{total}}=\lambda_{1}L_{\text{aux}}+\lambda_{2}L_{\text{pred}}+\lambda_{3}L_{\text{align}}, \end{array}$$

where $\lambda_{1}$, $\lambda_{2}$ and $\lambda_{3}$ are hyperparameters balancing the contributions of the structural robustness, primary prediction, and modality alignment, respectively.

## 3 Experiments and results

### 3.1 Datasets

The primary dataset is derived from (Sun *et al.* 2025), which integrates biochemical records from BRENDA (Schomburg *et al.* 2017) and SABIO-RK (Wittig *et al.* 2018) through established data processing protocols (Li *et al.* 2022a, Kroll *et al.* 2023). The dataset contains substrate SMILES, enzyme sequences, product SMILES, organism information, EC numbers, and experimentally measured $k_{\text{cat}}$ values. Due to substantial missingness in environmental annotations, pH and temperature information were excluded (Sun *et al.* 2025). We further augmented this dataset with protein textual descriptions from the function and catalytic activity fields. These annotations were generated by the Qwen3-Max Large Language Model (LLM) (Yang *et al.* 2025), leveraging the powerful capabilities of LLM in biomedical text understanding (Tian *et al.* 2024, Ai *et al.* 2026). In parallel, native annotations from UniProtKB were extracted from the uniprot_sprot.dat file, with missing entries replaced by a None token.

The final dataset comprises 26 713 entries, including 21 381 entries split into training and validation sets at a 9:1 ratio, with the remaining 5332 entries reserved for independent testing. Detailed statistics of the UniProtKB annotations are provided in Table S1.

### 3.2 Experimental setup

We evaluated Katalyst for enzyme $k_{\text{cat}}$ prediction using the multimodal dataset described in Section 3.1. The model was implemented in PyTorch and trained on an NVIDIA GeForce RTX 5060 Ti GPU. During training, we set the initial learning rate to $3\times 10^{-5}$ and utilized the cosine annealing scheduler for dynamic learning rate adjustment, while incorporating a dropout rate of 0.2 to prevent overfitting. To ensure robustness, we reported all results as the average over five independent runs with distinct random seeds, measured by Root Mean Squared Error (RMSE), Coefficient of Determination ($R^{2}$), and Spearman’s Rank Correlation Coefficient (SRCC). The loss weights ($\lambda_{1}=0.7$, $\lambda_{2}=0.2$, and $\lambda_{3}=0.1$) and contrastive temperature (τ=0.5) were fixed a priori and held constant throughout all experiments. Detailed hyperparameter configurations are provided in Table S2.

### 3.3 Results

#### 3.3.1 Text source evaluation and parameter sensitivity

Table 1 compares the performance of Katalyst across different textual sources, including LLM-generated corpus and expert-curated UniProtKB corpus, each constructed from the function and catalytic activity fields.

**Table 1:** Predictive performance across different textual semantic sources and dimensions.

Text Source	Semantic Field	**RMSE** ↓	$R^{2}↑$	**SRCC** ↑
Synthetic (LLM)	Catalytic activity	1.01795${}_{\pm 0.00338}$	0.55729${}_{\pm 0.00295}$	0.74055${}_{\pm 0.00113}$
Synthetic (LLM)	Function	1.01115${}_{\pm 0.00283}$	0.56319${}_{\pm 0.00244}$	0.74288${}_{\pm 0.00261}$
Native (UniProtKB)	Catalytic activity	0.99173${}_{\pm 0.00394}$	0.57980${}_{\pm 0.00333}$	0.75717${}_{\pm 0.00369}$
**Native (UniProtKB)**	**Function**	**0.98802** ${}_{\pm 0.00926}$	**0.58291** ${}_{\pm 0.00782}$	**0.76027** ${}_{\pm 0.00381}$

Mean values are presented with standard deviations shown as subscripts. Bold values indicate the best performance.

For the LLM-generated corpus, the function field achieved slightly better performance than the catalytic activity field, yielding an RMSE of 1.01115, an $R^{2}$ of 0.56319, and an SRCC of 0.74288, compared with an RMSE of 1.01795, an $R^{2}$ of 0.55729, and an SRCC of 0.74055 for the catalytic activity field. In contrast, the expert-curated corpus yielded substantial gains, with the function field achieving the best overall performance, including an RMSE of 0.98802, an $R^{2}$ of 0.58291, and an SRCC of 0.76027, followed by the catalytic activity field. These findings indicated that expert-curated biochemical annotations provided better semantic constraints than LLM-generated text, although the latter offered better scalability under sparse data conditions. Consequently, the UniProtKB function field was selected as the optimal textual feature for Katalyst. Meanwhile, the LLM-guided generative paradigm remains a valuable approach for future applications.

To assess model robustness, we conducted a sensitivity analysis by varying individual hyperparameters, including the loss weights $\left(\lambda_{1},\lambda_{2},\lambda_{3}\right)$ and the batch size. As validated by the results in Table S3, the model remained stable across different loss weight settings, with all evaluation metrics varying by approximately 1%. Increasing the batch size from 8 to 128 consistently improved performance, yielding the lowest RMSE of 0.98785 and the highest $R^{2}$ of 0.58308 at a batch size of 128. Although the SRCC exhibited minor fluctuations, the overall performance variation remained within approximately 0.4% across all batch sizes. These results indicate that Katalyst is robust to hyperparameter variations.

#### 3.3.2 Quantitative comparison of textual modality priors

To compare expert-curated semantic knowledge with LLM-generated semantic knowledge, we characterized the function descriptions in terms of text length, lexical diversity, and cross-modal alignment. We first analyzed the structural properties of enzyme function descriptions, as reported in Table 2 and Fig. S1. The expert-curated corpus showed a broad length distribution, with 48.50±47.66 words, reflecting diverse annotation patterns across protein families. By contrast, the LLM-generated corpus presented a more concentrated length distribution, with 39.42±7.33 words, consistent with the structured output style induced by instruction-guided generation (Zhang *et al.* 2026).

**Table 2:** Statistical analysis of function field descriptions across different sources.

Text Source	Text Length	TTR	MATTR
UniProtKB	**48.50** ${}_{\pm 47.66}$	0.8381${}_{\pm 0.1104}$	0.9169${}_{\pm 0.0596}$
LLM	39.42${}_{\pm 7.33}$	**0.8853** ${}_{\pm 0.0496}$	**0.9407** ${}_{\pm 0.0336}$

Data are presented as the empirical mean ± standard deviation. Bold values indicate the best performance.

We further quantified lexical diversity using Type-Token Ratio (TTR) (Templin 1957) and Moving-Average Type-Token Ratio (MATTR) (Covington and McFall 2010). The LLM-generated corpus showed TTR and MATTR values of 0.8853±0.0496 and 0.9407±0.0336, respectively. The expert-curated corpus showed corresponding values of 0.8381±0.1104 and 0.9169±0.0596, reflecting different lexical usage patterns between the two sources of semantic knowledge.

We evaluated cross-modal alignment by calculating the cosine similarity between text and sequence embeddings generated on the test set. These embeddings were extracted from Katalyst models trained with five independent random seeds (Fig. 2 and Table S4). The median alignment scores for expert-curated semantic knowledge were consistently higher and more tightly concentrated across different random seeds, remaining close to a stable baseline. In contrast, the LLM-generated semantic knowledge exhibited lower and more dispersed alignment distributions. A notable deviation was observed under Seed 4, where the LLM-generated semantic knowledge exhibited a transient increase in alignment scores that did not translate into improved predictive performance. To account for this phenomenon, we analyzed the intra-modal geometry by computing the average cosine similarity within the textual and protein sequence embedding spaces, respectively (Table S5). The LLM-generated text embeddings showed a more compact structure, with an average pairwise cosine similarity of 0.4791±0.1098. A similar pattern was observed in the protein sequence embeddings, where the similarity distribution reached 0.8056±0.0880. This spatial clustering increased cross-modal similarity but compromised the ability to distinguish fine-grained differences, suggesting a superficial alignment effect rather than precise semantic matching.

**Figure 2:**
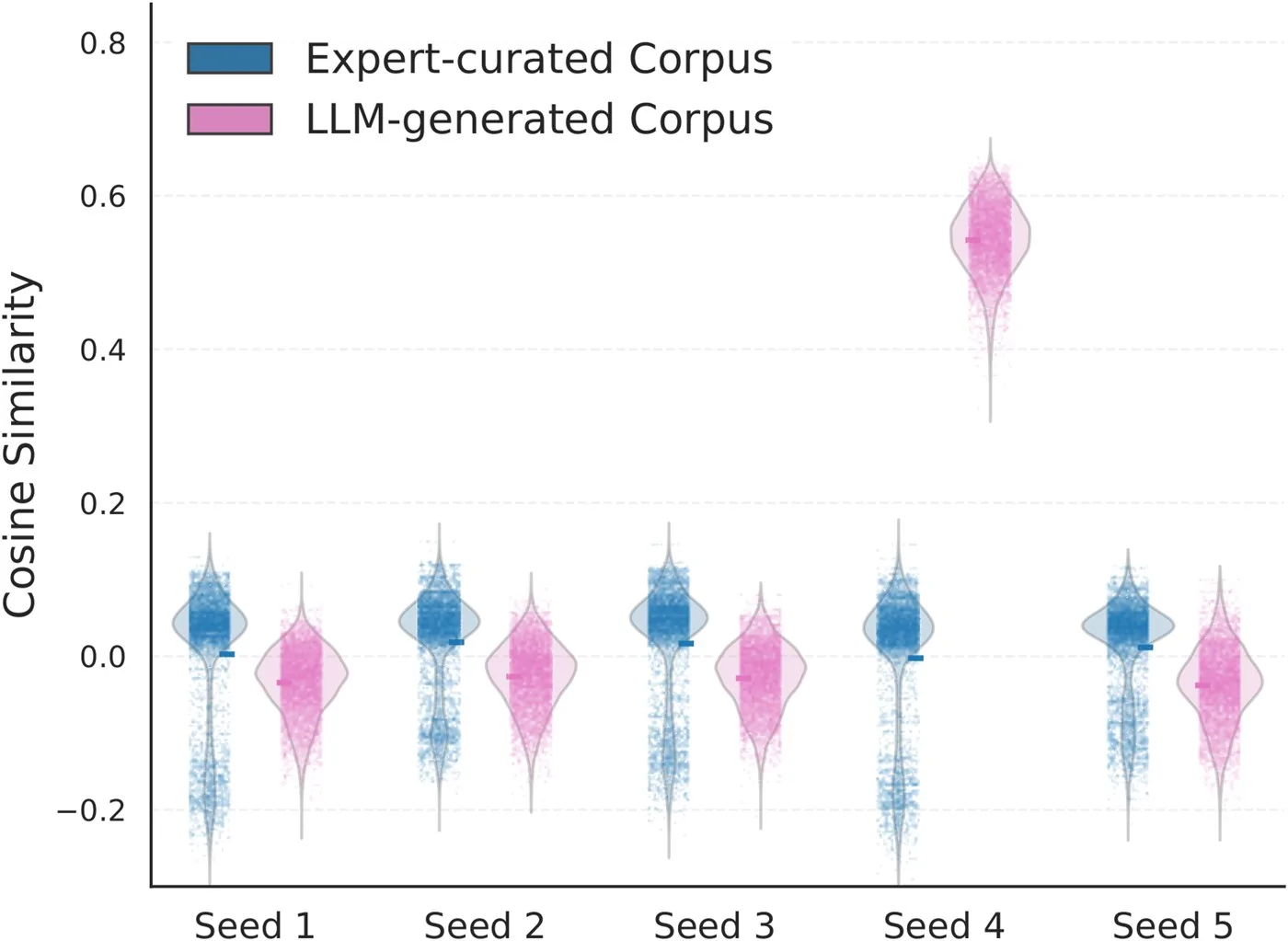
Cross-modal embedding alignment of function field representations across five independent training seeds. Protein sequence and function text embeddings were extracted from the adapter layers of Katalyst models independently trained with five random seeds (indexed as 1–5). Cosine similarity between paired sequence and text embeddings was calculated on the test set. Violin distributions represent the empirical density of alignment scores, with horizontal bars indicating the median values. Blue and pink violins represent the expert-curated corpus (from UniProtKB) and the LLM-generated corpus (generated by Qwen3-Max), respectively.

Overall, these results indicate a trade-off associated with LLM-based semantic knowledge augmentation. While the LLM-generated descriptions improve lexical diversity, their structural regularity may induce latent space collapse and unstable cross-modal alignment.

#### 3.3.3 Baselines

To evaluate Katalyst, we compared it with six SOTA $k_{\text{cat}}$ prediction frameworks, including DLKcat (Li *et al.* 2022a), DLTKcat (Qiu *et al.* 2023), EITLEM-Kinetics (Shen *et al.* 2024), UniKP (Yu *et al.* 2023), TurNuP (Kroll *et al.* 2023), and MMKcat (Sun *et al.* 2025). To ensure a rigorous and equitable comparison, all baselines were benchmarked under an identical data-splitting protocol, with their empirical performance reported as the mean ± standard deviation across five independent runs. Additionally, DLKcat, DLTKcat, EITLEM-Kinetics, and UniKP were designed for single-substrate inputs. Therefore, multiple substrates were concatenated using the dot delimiter, while product information was excluded. TurNuP natively supported multiple substrates and products, which were likewise concatenated with the dot delimiter and directly used as inputs. In MMKcat, substrates and products were encoded separately and aggregated via mean pooling. For models requiring assay conditions, missing assay conditions (e.g., pH and temperature) were set to zero.

As demonstrated in Fig. 3, Katalyst consistently outperformed all SOTA baselines across all evaluation metrics, establishing a new performance benchmark. Specifically, Katalyst achieved the lowest mean RMSE of 0.98802, the highest $R^{2}$ of 0.58291, and the highest SRCC of 0.76027. Compared to the most competitive baseline, MMKcat, which also utilized multimodal information, our model exhibited a 3.01% reduction in RMSE, alongside relative improvements of 4.72% in $R^{2}$ and 2.31% in SRCC. Furthermore, Katalyst maintained a significant performance margin over EITLEM-Kinetics, the next most competitive baseline in our study. Specifically, relative to EITLEM-Kinetics, our model achieved a 3.36% reduction in RMSE and substantial improvements of 5.33% in $R^{2}$ and 3.22% in SRCC. These results further highlighted the ability of Katalyst to provide more accurate and robust predictions of $k_{\text{cat}}$ compared to other models across diverse reaction spaces.

**Figure 3:**
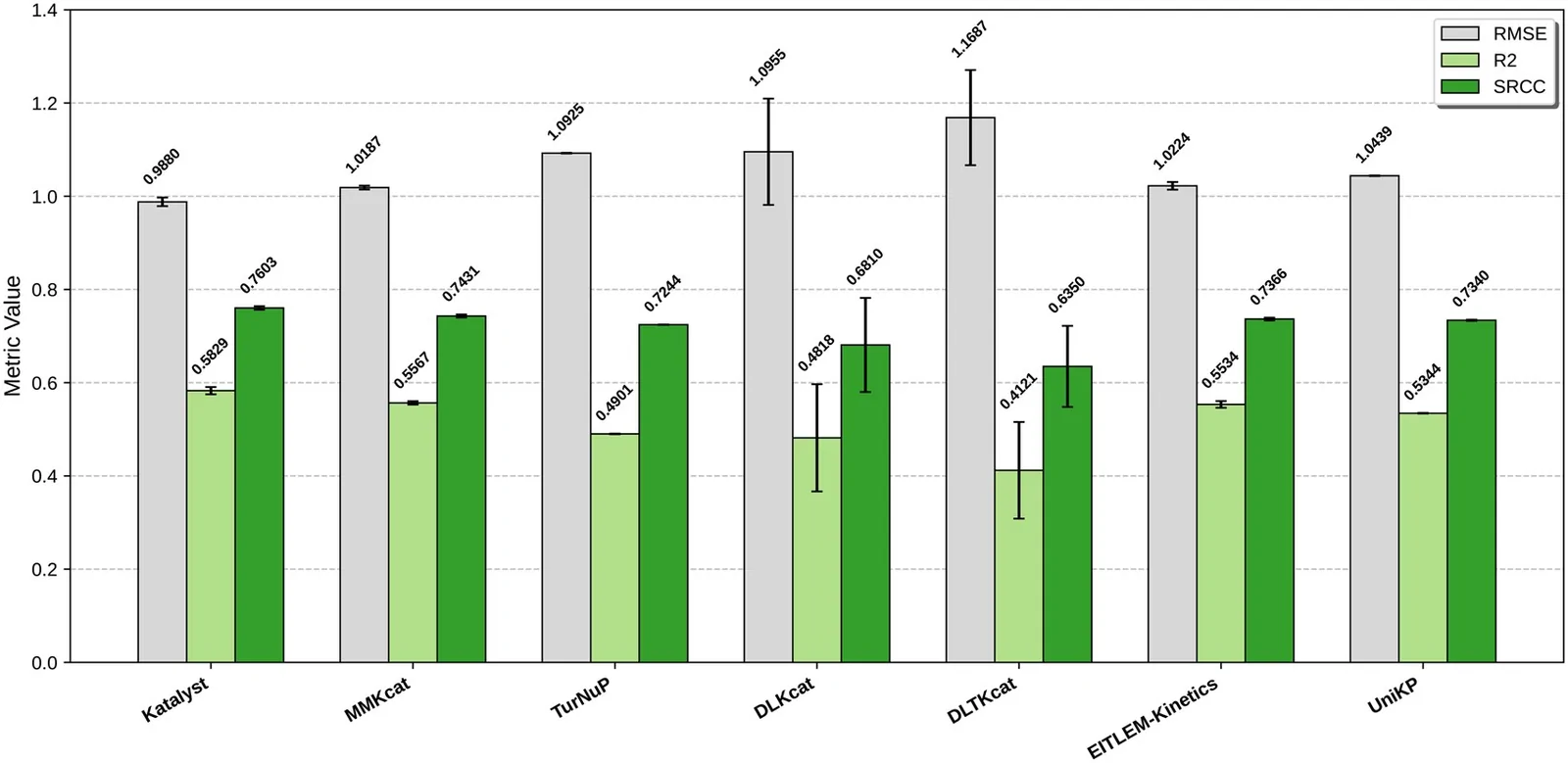
Comprehensive performance comparison of Katalyst and baseline models. The grouped bar chart illustrates the mean values for RMSE, $R^{2}$, and SRCC, with error bars representing the standard deviation across five independent runs.

#### 3.3.4 Ablation study

To systematically investigate the individual contributions of various modules, we performed comprehensive ablation experiments and evaluated five ablated variants: (1) w/o Text, which completely excluded textual input; (2) w/o Fusion, which removed the late stage feature fusion module; (3) w/o Cross-Modal Contrast, which omitted contrastive alignment across all modalities, including both the text-to-enzyme alignment and the enzyme-substrates-products alignment; (4) w/o Text-Guided Contrast, which removed the contrastive learning between text descriptions and enzyme sequences; and (5) w/o Tri-Entity Contrast, which specifically disabled alignment between enzyme sequences, substrate, and product representations.

Ablation results in Table 3 demonstrate the contribution of each component in Katalyst. The full model achieved the best SRCC of 0.76027, indicating superior ranking consistency, while maintaining a competitive RMSE of 0.98802 and an $R^{2}$ of 0.58291. The most significant degradation was observed in the w/o Fusion variant, where both RMSE and SRCC worsened markedly to 1.00936 and 0.74124, highlighting the importance of multimodal integration. Removing text information or contrastive learning modules (w/o Text, w/o Cross-Modal Contrast, and w/o Text-Guided Contrast) also consistently reduced performance, confirming the benefit of semantic and alignment constraints. Notably, the w/o Tri-Entity Contrast variant yielded marginal improvements, with an RMSE of 0.98796 and an $R^{2}$ of 0.58298. However, these gains were marginal and came at the cost of a lower SRCC, suggesting a trade-off between accuracy and ranking consistency. Consequently, the complete architecture achieved the most robust trade-off, sustaining virtually optimal regression precision while maximizing ranking consistency.

**Table 3:** Ablation studies of Katalyst.

Method	**RMSE** ↓	$R^{2}↑$	**SRCC** ↑
w/o Text	0.99121${}_{\pm 0.0045}$	0.58024${}_{\pm 0.0038}$	0.75544${}_{\pm 0.0025}$
w/o Fusion	1.00936 ${}_{\pm 0.0025}$	0.56474${}_{\pm 0.0022}$	0.74124${}_{\pm 0.0014}$
w/o Cross-Modal Contrast	0.98901${}_{\pm 0.0050}$	0.58210${}_{\pm 0.0043}$	0.75732${}_{\pm 0.0029}$
w/o Text-Guided Contrast	0.99021${}_{\pm 0.0079}$	0.58107${}_{\pm 0.0067}$	0.75638${}_{\pm 0.0055}$
w/o Tri-Entity Contrast	0.98796${}_{\pm 0.0062}$	0.58298${}_{\pm 0.0052}$	0.75788${}_{\pm 0.0047}$
Katalyst	0.98802${}_{\pm 0.0093}$	0.58291${}_{\pm 0.0078}$	0.76027${}_{\pm 0.0038}$

#### 3.3.5 Impact of protein sequence length

To explore the performance of the model across protein sequences of varying lengths, we evaluated its behavior over 20 sequence length groups ranging from 12 to 720 amino acids, as summarized in Fig. 4. Katalyst maintained a stable RMSE between 0.80 and 1.01 across most intervals, specifically from Group 1 to 3 and Group 8 to 19. Notably, for exceptionally long sequences in Group 19, Katalyst constrained the RMSE to 1.01, whereas Katalyst (w/o Text) variant degraded sharply to greater than 1.6, demonstrating that professional function field annotations provided critical semantic constraints to stabilize the latent space. In terms of ranking, Katalyst exhibited a consistently high SRCC from Group 3 to 17. The sharp SRCC decline observed in Group 20 was a statistical artifact of data sparsity (n=2), where rank correlation became hypersensitive to minor fluctuations, rather than a failure of architectural generalization. These results indicated that Katalyst robustly captured biochemical trends across diverse sequence scales.

**Figure 4:**
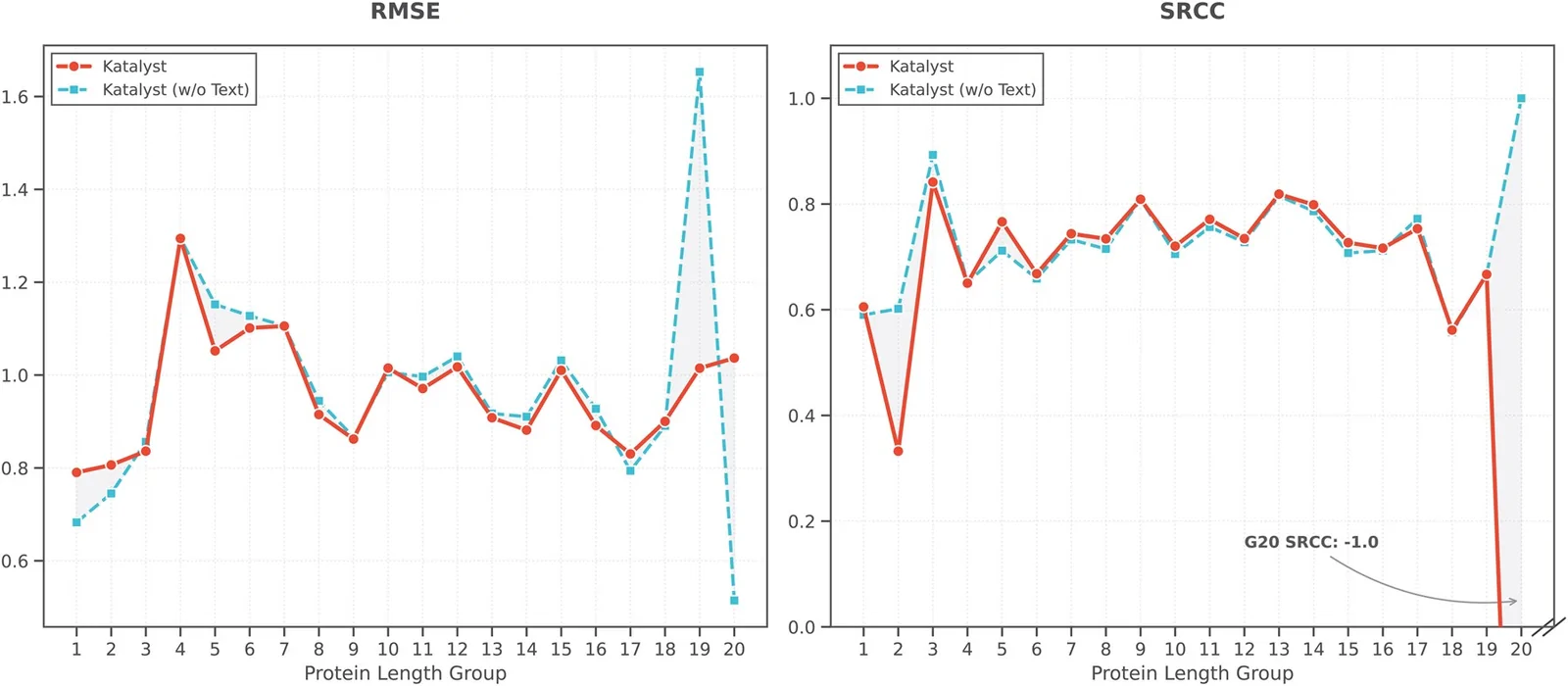
Predictive performance across varying enzyme sequence lengths. The test set is partitioned into 20 groups based on protein length, with RMSE and SRCC metrics compared between Katalyst and its variant, Katalyst (w/o Text). Katalyst demonstrates superior error control, particularly in the stable range of Groups 8 to 19 and the shortest sequences (Groups 1 to 3). Notably, Katalyst maintains consistent ranking performance (SRCC) across the broad interval of Groups 3 to 17 and successfully mitigates the RMSE performance collapse observed in Katalyst (w/o Text) variant for exceptionally long sequences (Group 19). Data points represent the mean values, illustrating the robust stabilization provided by professional functional annotations across diverse protein scales. Note that the SRCC values for Group 20 reflect statistical fluctuations due to limited sample size (n=2).

#### 3.3.6 Case study

To intuitively assess predictive performance, we evaluated the absolute error, defined as $\left|k_{\text{cat}}^{\text{pred}}-k_{\text{cat}}^{\text{true}}\right|$, across representative catalytic reactions against several SOTA baselines (Table 4). As tabulated, Katalyst demonstrated a distinct advantage in predictive accuracy, securing the minimum absolute error in the majority of representative cases (including Case 1, Case 2, and Case 5) and validating its exceptional capacity for multimodal biochemical integration.

**Table 4:** Comparison of absolute errors between predicted and true $k_{\text{cat}}$ values across representative cases.

Case Index	Case 1	Case 2	Case 3	Case 4	Case 5
	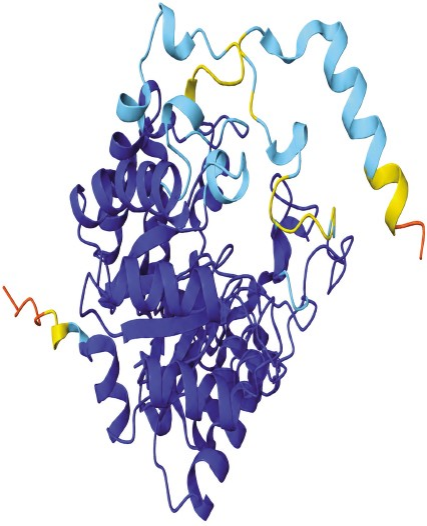	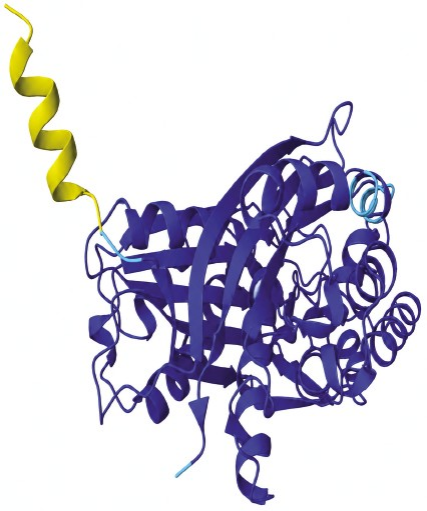	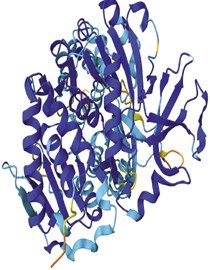	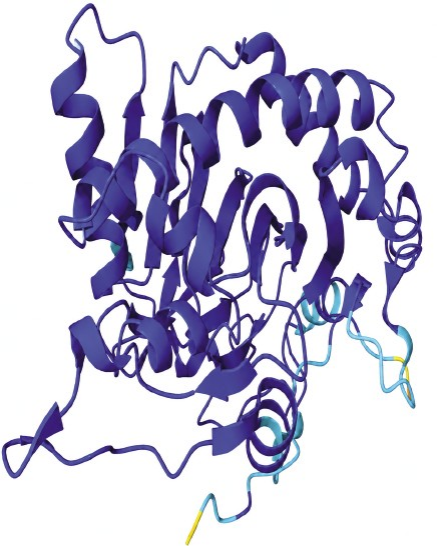	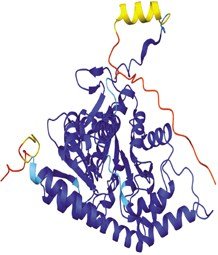
UniProt ID	O09171	L7NCQ3	Q6TNA5	Q96330	Q9ZQI7
	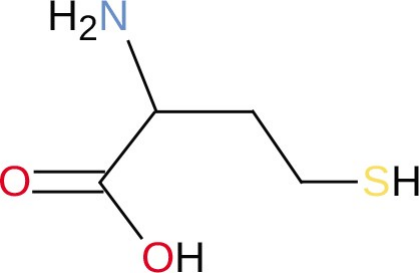	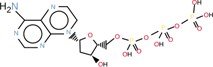	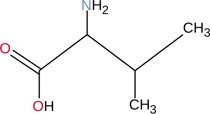	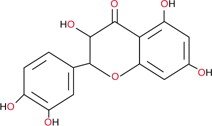	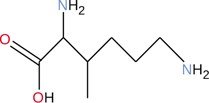
SMILES	C(CS)C(C(=O)O)N	CC(C)(COP(=O)(O) OP(=O)(O)OCC1C (C(C(O1)N2C = NC3 =C(N = CN = C32)N) O)OP(=O)(O)O)C (C(=O)NCCC(=O)N CCSC(=O)C4 = CC = CC = C4)O	CC(C)C(C(=O)O)N	C1 = CC(=C(C = C1C2C(C(=O)C3 = C(C = C(C = C3O2)O)O)O)O)O	C(CCN)CC(C(=O)O)N
**Katalyst**	**0.00063**	**0.00056**	**0.00128**	0.00142	**0.00875**
**Katalyst (w/o Text)**	0.00073	0.01228	0.05698	0.01747	0.16860
MMKcat	0.00536	0.02629	0.00425	0.01689	1.31024
EITLEM-Kinetics	0.00372	0.03455	0.02954	**0.00059**	0.34215
UniKP	0.00215	0.06724	0.00649	0.02054	0.04594
TurNuP	0.10012	0.06763	0.96837	0.04475	0.36564
DLTKcat	1.00449	0.09590	7.42382	0.00241	0.59079
DLKcat	0.00335	0.05272	0.00502	0.00213	0.40840

Bold values indicate the smallest absolute values (best performance). Underlined values indicate the second-best performance.

Notably, even in the absence of textual functional annotations, the Katalyst (w/o Text) variant consistently maintained high precision, frequently ranking among the top three performers. This robust performance underscored that while professional function descriptions further refined predictions to an optimal level, our core architecture inherently excelled at extracting critical catalytic features and intricate protein-ligand interactions from sequence and structural data alone.

## 4 Conclusion

In this study, we developed Katalyst, a multimodal representation framework that utilizes multi-stage contrastive alignment and an interaction-driven fusion module integrating MHA and CNN architectures to enhance the accuracy and robustness of $k_{\text{cat}}$ prediction. Comparative analysis between LLM-generated semantic knowledge and expert-curated semantic knowledge revealed that expert-curated biological knowledge provided better semantic constraints. Nevertheless, LLM-guided biological text generation remains a valuable approach for future applications, with future improvements dependent on biological knowledge integration and enhanced cross-modal alignment for scalable functional representation learning (Kulmanov *et al.* 2021). Based on these robust cross-modal alignment foundations, extensive benchmarking demonstrated that Katalyst consistently outperformed SOTA baselines, setting a new performance benchmark with a 3.01% reduction in RMSE, alongside 4.72% and 2.31% improvements in $R^{2}$ and SRCC, respectively. Ablation studies and evaluations across sequence scales further confirmed the architectural robustness of Katalyst.

Nevertheless, the framework has limitations, including the lack of explicit consideration of environmental and reaction-condition factors and the hallucination risks associated with LLM-generated semantic knowledge, as inaccurate or overly generalized descriptions may introduce noise into semantic representations. Future work will further improve LLM-based semantic generation through biological constraints and expert validation to enhance the reliability of functional descriptions. Moreover, we will incorporate broader environmental factors and extend the framework to the engineering of novel biocatalysts.

## Supplementary Material

btag709_Supplementary_Data

## Data Availability

The data underlying this article are available in https://github.com/liruilin-SUAT/Katalyst.
