# Supplementary material for: Katalyst: knowledge-guided semantic alignment via contrastive learning for enzyme turnover prediction
Source: Bioinformatics. 2026 Oct 3;42(10):btag709. doi: 10.1093/bioinformatics/btag709 (PMC13649181; doi:10.1093/bioinformatics/btag709)
Supplement: Supplementary file 1 [file btag709_supplementary_data.pdf]

Supplementary Material

Supplementary Table S1: Statistics of Missing Annotations (None Tokens) in the Dataset

**Annotation Field**	**Dataset Subset**	**Total Entries**	`None` **Entries**	**Missing Ratio (%)**
function	train/validation set	21,381	4,286	20.05%
	test set	5,332	1,057	19.82%
catalytic activity	train/validation set	21,381	2,356	11.02%
	test set	5,332	590	11.07%

Supplementary Table S2: Hyperparameter configurations for Katalyst.

**Hyperparameter**	**Value**	**Description**
batch size	32	Number of samples per training batch
learning rate (lr)	3×10^{-5}	Initial learning rate
dropout	0.2	Probability for dropout layers
attention heads ($nhead$)	8	Number of heads in Multi-Head Attention
temperature (τ)	0.5	Scaling factor for InfoNCE
λ_1	0.7	Weight coefficient for the auxiliary predictor loss
λ_2	0.2	Weight coefficient for the k_{cat} predictor loss
λ_3	0.1	Weight coefficient for the contrastive alignment loss

Supplementary Table S3: Parameter sensitivity analysis of Katalyst via controlled single-parameter variations. Each section presents results where the specified hyperparameter is systematically varied while keeping all other architectural configurations strictly at their default values. The best results within each investigated category are highlighted in **bold**(mean ± standard deviation across five independent random seeds).

**Configuration / Value**	**RMSE** ↓	R^2 ↑	**SRCC** ↑
Block 1: Multi-task Loss Weight Distribution $(\lambda_1, \lambda_2, \lambda_3)$			
Variation on λ_1 *(Auxiliary Regularization)*			
$(\lambda_1 = \mathbf{0.6}, \lambda_2 = 0.2, \lambda_3 = 0.1)$	0.98948 ± 0.00487	0.58171 ± 0.00412	**0.76037 ± 0.00212**
$(\lambda_1 = \mathbf{0.7}, \lambda_2 = 0.2, \lambda_3 = 0.1)$ (**Default**)	0.98802 ± 0.00926	0.58291 ± 0.00782	0.76027 ± 0.00381
$(\lambda_1 = \mathbf{0.8}, \lambda_2 = 0.2, \lambda_3 = 0.1)$	**0.98663 ± 0.00609**	**0.58411 ± 0.00512**	0.76013 ± 0.00232
Variation on λ_2 *(Primary Regression)*			
$(\lambda_1 = 0.7, \lambda_2 = \mathbf{0.1}, \lambda_3 = 0.1)$	0.98847 ± 0.00915	0.58253 ± 0.00774	0.75813 ± 0.00653
$(\lambda_1 = 0.7, \lambda_2 = \mathbf{0.3}, \lambda_3 = 0.1)$	0.99255 ± 0.00766	0.57909 ± 0.00650	0.75438 ± 0.00600
Variation on λ_3 *(Contrastive Alignment)*			
$(\lambda_1 = 0.7, \lambda_2 = 0.2, \lambda_3 = \mathbf{0.2})$	0.98971 ± 0.00650	0.58150 ± 0.00548	0.75994 ± 0.00306
$(\lambda_1 = 0.7, \lambda_2 = 0.2, \lambda_3 = \mathbf{0.3})$	0.98853 ± 0.00573	0.58251 ± 0.00483	0.76019 ± 0.00196
Block 2: Contrastive Learning Batch Size (B)			
$B = 8$	0.99403 ± 0.00669	0.57784 ± 0.00570	0.75842 ± 0.00283
$B = 16$	0.98987 ± 0.00615	0.58137 ± 0.00519	0.75943 ± 0.00219
$B = 32$ (**Default**)	0.98802 ± 0.00926	0.58291 ± 0.00782	**0.76027 ± 0.00381**
$B = 64$	0.98858 ± 0.00440	0.58247 ± 0.00371	0.75868 ± 0.00090
$B = 128$	**0.98785 ± 0.00350**	**0.58308 ± 0.00296**	0.75740 ± 0.00162

Supplementary Table S4: Cross-modal embedding alignment across five data-splitting configurations. To evaluate robustness, five random seeds (indexed as 1–5) were used exclusively to partition the training and validation sets. Five Katalyst models were independently trained on these respective data splits. Sequence and text embeddings were then extracted from their adapter layers to compute pairwise cosine similarities on the test set (reported as mean ± standard deviation).

**Data-Splitting (Seed)**	**Expert-curated Corpus**	**LLM-generated Corpus**
1	0.0027 ± 0.0898	-0.0343 ± 0.0435
2	0.0183 ± 0.0653	-0.0265 ± 0.0434
3	0.0167 ± 0.0770	-0.0286 ± 0.0426
4	-0.0025 ± 0.0908	$\mathbf{0.5421 \pm 0.0468}$
5	0.0116 ± 0.0634	-0.0378 ± 0.0488

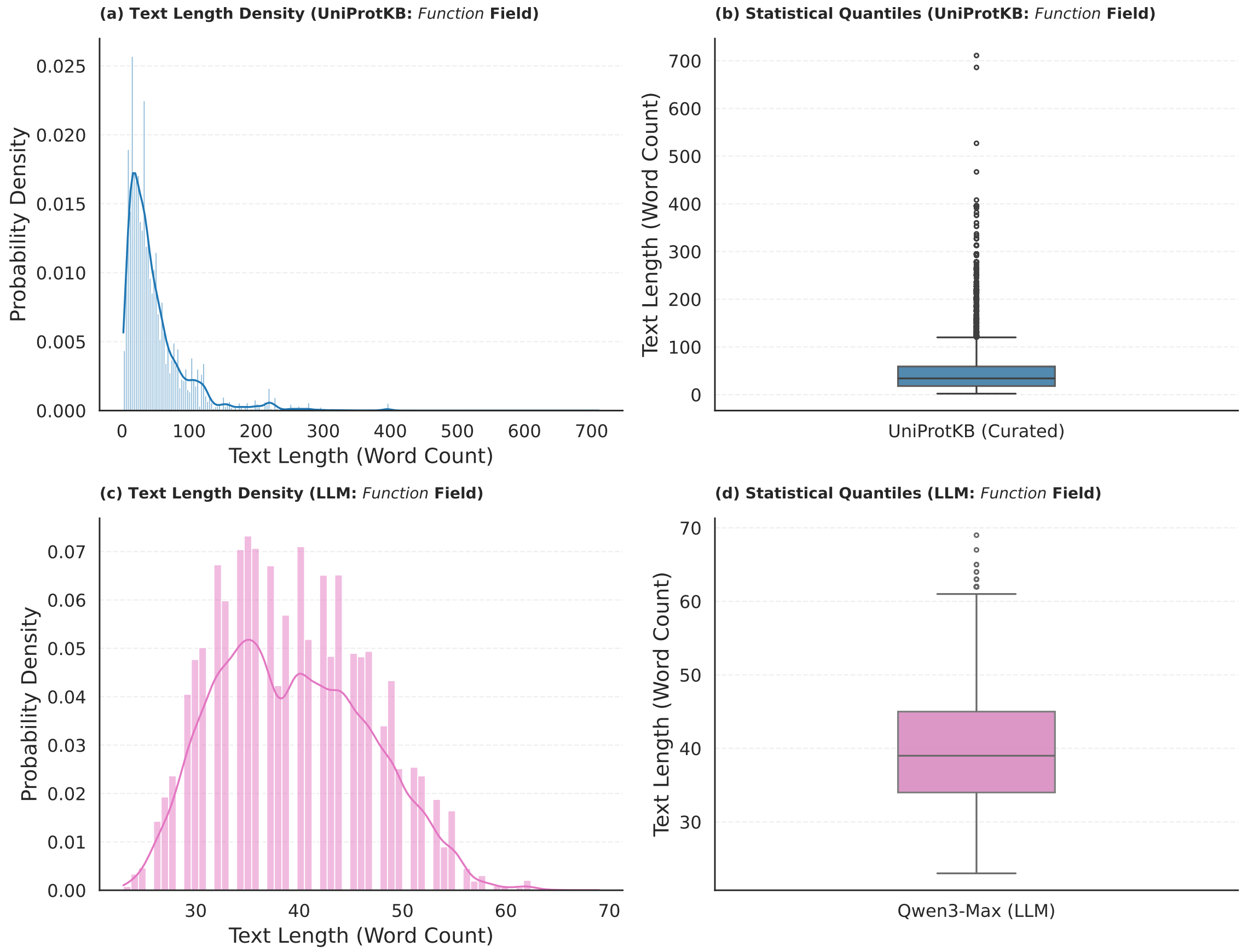

Supplementary Figure S1: Empirical length distributions of protein functional field descriptions across expert-curated and LLM-generated corpora. The analysis characterizes structural differences based on word counts per protein entry. (a) Empirical probability density function and kernel density estimation curve for the expert-curated corpus from UniProtKB. (b) Statistical quantile boxplot for the expert-curated corpus. (c) Empirical probability density function and kernel density estimation curve for the LLM-generated corpus from Qwen3-Max. (d) Statistical quantile boxplot for the LLM-generated corpus.

Supplementary Table S5: Anisotropy analysis of intra-modal latent manifold structures. Reported values denote the empirical mean ± standard deviation of pairwise cosine similarities evaluated on the test set. Five unique random seeds (indexed as 1–5) were employed exclusively to control the random partition of the training and validation sets. Five independent models were then trained on these respective data splits, from which text and biological sequence embeddings were extracted for intra-modal similarity analysis.

Data-Splitting (Seed)	Expert-curated Corpus		LLM-generated Corpus	
	Text-to-Text	Seq-to-Seq	Text-to-Text	Seq-to-Seq
1	0.1719 ± 0.2018	0.6179 ± 0.1074	0.1693 ± 0.1201	0.7132 ± 0.0833
2	0.1637 ± 0.2031	0.6732 ± 0.0866	0.1741 ± 0.1190	0.7170 ± 0.0841
3	0.1586 ± 0.2056	0.5949 ± 0.1117	0.1800 ± 0.1222	0.7120 ± 0.0814
4	0.1614 ± 0.2002	0.6327 ± 0.1061	$\mathbf{0.4791 \pm 0.1098}$	$\mathbf{0.8056 \pm 0.0880}$
5	0.1735 ± 0.1994	0.6530 ± 0.1046	0.1882 ± 0.1149	0.6694 ± 0.1021
